# Efficacy of Paracetamol in Closure of Ductus Arteriosus in Infants under 32 Weeks of Gestation

**DOI:** 10.3389/fped.2018.00025

**Published:** 2018-02-14

**Authors:** Ines Tofe, Maria Dolores Ruiz-González, Maria Dolores Cañete, Asuncion Pino, Rosa Lorena Rueda, Maria Jose Parraga, Juan Luis Perez-Navero

**Affiliations:** ^1^Hospital Reina Sofía de Córdoba, Cordova, Spain; ^2^Instituto Maimonides de Investigación Biomédica de Cordoba (IMIBIC), Cordova, Spain; ^3^Pediatrics, Hospital Alto Guadalquivir, Andújar, Spain

**Keywords:** patent ductus arteriosus, paracetamol, preterm, treatment, ibuprofen

## Abstract

**Background:**

Standard medical treatment for patent ductus arteriosus (PDA) closure has been indomethacin/ibuprofen or surgical ligation. Up to date, new strategies have been reported with paracetamol. The aim of this study was to present our experience with intravenous paracetamol for closing PDA in preterm neonates presenting contraindication to ibuprofen or ibuprofen had failed and no candidates for surgical ligation because of huge instability.

**Materials and methods:**

We conducted a retrospective case series study in a neonatal intensive care unit from a tertiary hospital. 9 preterm infants ≤32 weeks of gestational age with hemodynamically significant PDA (hsPDA) were enrolled. They received 15 mg/kg/6h intravenous paracetamol for ductal closure. Demographic data and transaminase levels before and after treatment were collected.

**Results:**

30 preterm babies were diagnosed of hsPDA. 11/30 received ibuprofen with closure in 81.1%. 9 received intravenous paracetamol mainly due to bleeding disorders or thrombocytopenia. Successful closure on paracetamol was achieved in seven of nine babies (77.7%). There was a significant increase in transaminase levels in two patients. They required no treatment for normalization.

**Conclusion:**

Paracetamol is an effective option in closure PDA. It should be a first-line therapeutic option when there are contraindications for ibuprofen treatment. Transaminases must be checked during treatment.

## Introduction

Closure of ductus arteriosus after birth is very important for circulation adaptation to the extrauterine life. Patent ductus arteriosus (PDA) in extremely premature infants is associated with morbidities such as necrotizing enterocolitis, bronchopulmonary dysplasia (BPD), and neurodevelopmental disabilities (1). Standard medical treatment for PDA closure has been indometacin/ibuprofen or surgical ligation. Adverse events have been reported with NSAIDs (2), and surgical ligations have been associated with a higher incidence of BPD, retinopathy of prematurity, and neurodevelopmental disorders (3).

Hammerman et al. reported for the first time the use of paracetamol for closing PDA (4). Since then, many studies have reported similar efficiency of paracetamol to COX-inhibitors for closing PDA and less adverse events (5).

The aim of this study was to present our experience with intravenous (iv) paracetamol for closing PDA in preterm neonates presenting contraindication to ibuprofen or ibuprofen had failed and had feeding intolerance.

## Materials and Methods

We conducted a retrospective case series study of 30 preterm infants of ≤32 weeks of gestational age (GA) with hemodynamically significant PDA (hsPDA) from May 2015 to January 2017. The medical records were retrospectively evaluated. We collected the percentage of spontaneous closure, the percentage of patients who received ibuprofen versus paracetamol or surgical ligation. Ibuprofen was given intravenously at a regimen of 10, 5, and 5 mg/kg/day [for 3 days, respectively. *Pedea* (orphan drug) 5 mg/ml]. Nine premature infants received paracetamol (Paracetamol B. Braun 10 mg/ml), 15 mg/kg iv administration every 6 h. All had hsPDA clinically diagnosed and confirmed by means of echo-Doppler cardiography.

Echocardiography criteria of hsPDA were a ductal diameter ≥1.5 mm, a left atrium to aortic root ratio >1.5, and diastolic aortic retrograde flow. Bidimensional color Doppler echocardiography with Philips HD7 GE Healthcare multifrequency 8 MHz sector probe was used. Daily echocardiographic examination was conducted. If ductus closure was confirmed by echocardiography, treatment was discontinued. The study was carried out in accordance with the recommendations of RSUH Ethics and Research Committee.

Primary reason for using paracetamol was failure to response to ibuprofen administration or the presence of absolute contraindications for ibuprofen (bleeding, platelet count ≤ 60,000, intraventricular hemorrhage, and pulmonary hemorrhage).

Demographic features (GA, gender, birth weight, height, head circumference, Apgar score, delivery mode, antenatal steroids, MgSO_4_, age treatment/days of treatment, primary reason to use paracetamol, main outcome, adverse events, surgery, and invasive ventilation), antenatal exposure to steroids and magnesium sulfate, postnatal age at diagnosis, age at first paracetamol dose, duration of treatment, response to treatment, and need of surgical ligation were noted. Before and 24 h after the end of paracetamol treatment, liver function tests were performed in all patients. In all cases, a written informed consent was obtained.

## Results

Between May 2015 and January 2017, there were a total of 30 preterm infants who had significant PDA. 11/30 received ibuprofen, and 3/30 had a spontaneous closure (10%) with GA 28, 28, and 29 weeks. 7 died (4 under ibuprofen treatment, two received no treatment, and one underwent PDA ligation), and nine patients received iv paracetamol (5 of them of ≤28 weeks of GA). Results among the nine patients who underwent paracetamol were as follows: mean GA was 28 weeks ranging from 25 to 32 weeks and mean birth weight was 1,052 g ranging from 560 to 1,860 g. 3 preterm were male. All the patients had received antenatal steroids, and five of them have been exposure to antenatal magnesium sulfate as neuroprotection. Table 1 describes main clinical findings among infants who received paracetamol.

**Table 1:** Demographic characteristics among infants treated with paracetamol and main outcomes.

	GA (w)	Gender	Birth weight (g)	Height (cm)	Head circumference (cm)	Apgar 1′/5′	Delivery mode (C/V)	Antenatal steroids	MgSO_4_ pre	Age treatment/days of treatment	Primary reason use paracetamol	Main outcome	Adverse events	Surgery	Invasive ventilation
1	28	M	716	33.5	23	8/8	C	1	Yes	7/4	Thrombocytopenia, bleeding	No closed	No	Yes	Yes
2	29	M	1,000	35	26	7/9	C	1	No	4/6	Bleeding	Closed	No	No	Yes
3	25	F	560	31	22	4/7	C	1	Yes	4/6; 7/6	Fail ibuprofen, intraventricular hemorrhage	No closed (2 courses)	No	Yes	Yes
4	29	F	1,120	37	26	6/7	C	1	Yes	2/3	Intraventricular hemorrhage	Closed	No	No	Yes
5	27	F	930	38	24	5/7	C	Partial	No	2/3; 16/3	Intraventricular hemorrhage	Closed (2 courses)	No	No	Yes
6	31	M	1,860	42	32	5/6	C	0	No	3/2	Thrombocytopenia, coagulopatía	Closed	AST↑ ALT↑	No	Yes
7	32	F	1,495	41	29	7/8	C	1	No	5/3	Thrombocytopenia, pulmonary hemorrhage	Closed	AST↑	No	Yes
8	28	F	1,060	36	26	7/9	C	2 courses	Yes	35/6	Thrombocytopenia	Closed	No	No	Yes
9	26	F	731	30	22.5	4/7	C	1	Yes	3/3	Thrombocytopenia, intraventricular hemorrhage	Closed	No	No	Yes

*Echocardiography criteria of hsPDA were a ductal diameter ≥1.5 mm, a left atrium to aortic root ratio >1.5, and diastolic aortic retrograde flow. Bidimensional color Doppler echocardiography with Philips HD7 GE Healthcare multifrequency 8 MHz sector probe was used*.

*C, cesarean section; hsPDA, hemodynamically significant patent ductus arteriosus; V, vaginal delivery*.

In all patients, due to feeding intolerance and clinic instability, iv paracetamol was started after obtaining informed consent signature. Complete closure was observed in 7/9 (77.7%). The mean postnatal age at the first iv paracetamol dose was 4 days, ranging from 2 to 35 days. In eight of nine patients, the treatment was started in the first week of life. In eight infants, ibuprofen was contraindicated, and in one of them, the ibuprofen treatment had failed. One patient was treated for 2 days due to an elevation in liver transaminases, but ductus was closed so treatment was discontinued. Values were normalized 5 days later. Three patients were treated for 3 days, one for 4 days, and 2 for 6 days. Two patients needed two courses; one of them received acetaminophen for 3 days. In that case, ductus persisted opened even though ibuprofen was administered in a second course because there were no contraindications. Finally, baby went to surgical ligation on 16th day of life. One patient was successfully treated on the third day of life, but on the 16th day of life, ductus was reopened and a second course was conducted with definitively ductal closure after 48 h.

Table 2 shows transaminases levels before and after treatment with paracetamol.

**Table 2:** Liver tests before and 24 h after the end of paracetamol treatment.

	AST (U/L) before	AST (U/L) after	ALT (U/L) before	ALT (U/L) after
1	31	34	8	10
2	27	24	6	7
3	6/9	10/6	20/24	39/26
4	17	13	25	26
5	30/20	16/41	6/36	11/31
6	41	2,624	7	355
7	34	117	8	10
8	177	166	67	68
9	28	21	7	13

## Discussion

Hammerman et al. reported for the first time several case reports on premature infants who received paracetamol achieving ductal closure (4). Since then, 24 case reports series have been reported and 6 randomized control trials (RCTs) showing paracetamol utility for ductal closure with similar results comparing to ibuprofen/indomethacin and fewer adverse events.

Prostaglandins are relevant in PDA. Indomethacin and ibuprofen inhibit cyclooxygenase (COX3) in a no selective manner. How paracetamol acts for closing PDA still remains unclear, but it is known that it inhibits prostaglandin synthetase (5). Alternatively, paracetamol has been proposed to selectively inhibit a central isoform of COX3, but the existence of a functional human COX3 has been questioned (6).

Oncel et al. used paracetamol in 10 premature infants under than 30 weeks of GA with a 100% of effectiveness. Nevertheless, other authors did not achieve same striking results (7–9). The most common dosage is 15 mg/k/dose/q6h.

A report on high level of transaminases caused by iv paracetamol treatment for PDA closure in premature infants found that a lower dose of paracetamol also is effective (10, 11). Therefore, the dose and dose interval of iv paracetamol treatment might require revision.

One of our patient received paracetamol on day 35 of life and ductus was closed, even though the most studies state that the earliest beginning of treatment is the most effective. Some studies reported up to a 71.6% of ductal closure when it is administered after 20 days of life (11–17).

Among 13 observational studies published, paracetamol was orally given (18–22) (112 premature infants), and in 12 observational studies, paracetamol was given iv (150 premature infants) with a closing average up to a 72.2% in oral route versus 66% with iv paracetamol. Of the six RCTs, just one compared indomethacin and ibuprofen (9, 23–25) with no statistical significant differences. We have reported a higher average ductal closure probably because paracetamol was given earlier, in the first week of life. Only one patient had received ibuprofen first. Other observational studies show that paracetamol is more effective when there has been no exposure to ibuprofen and less effective when it is administered later (23, 26). In fact, one of our patients with no ductal closure after paracetamol who underwent surgical ligation had received ibuprofen first. Average of spontaneous ductal closure is higher at higher GAs. The small sample size is a limitation for our study, and four of nine patients who received paracetamol were of ≥29 weeks of GA. Nonetheless, the median GA among our 30 premature infants with hsPDA and spontaneous closure was 28 weeks, so we truly believe that the spontaneous closure among our population cannot be due to higher GAs.

The single adverse event we noticed was a transient elevation in liver enzymes in two patients as previously has been reported in literature, and they required no treatment.

Our patients had oral feeding intolerance, so we use iv route. In our opinion, the oral route probably does not represent the optimal choice for ELBW infants. In these patients, gut immaturity together with oral feeding intolerance typical of ELBW can lead to unpredictable and possibly too low intestinal drug absorption.

Since 2012, MgSO_4_ (magnesium sulfate) have been used as a neuroprotector agent among premature infants under 31 + 6 weeks of GA. 5 out of nine premature infants received MgSO_4_ as neuroprotection. del Moral et al. (27) related prenatal exposure to MgSO_4_ with higher incidence of hemodynamically significant persistent ductus arteriosus. Functional closure of the ductus after birth is primarily due to smooth muscle constriction, owing an increase in intracellular calcium concentration (27). Magnesium acts as a calcium antagonist, blocking calcium ion entry into the smooth muscles. More studies are probably needed to investigate the relationship between prenatal magnesium sulfate and PDA in premature infants. Some epidemiological studies suggest a link between early exposure to paracetamol and risk of asthma and other atopic diseases (28). Moreover, one study among 64,322 infants whose mother received acetaminophen during pregnancy reported a higher incidence of attention deficit syndrome and hyperactivity (29).

## Conclusion

Our results highlight that paracetamol could become not only an alternative treatment in closing PDA but also the treatment of choice in several scenarios. Nevertheless, one of the main limitations of this study is that it is a case series report with fewer subjects. More studies are needed to know long-term consequences of using paracetamol for closing PDA and to answer important questions about the optimal dose, the best route of administration, safety and the implications for neurodevelopmental, and long-term consequences.

## Author Contributions

IT: patient recruitment after informed consent was signed up, review of the charts, review of the literature, and writing of the manuscript. MR: review literature and patient recruitment and made corrections. MC: establish the protocol and writing of the special informed consent for an off-label indication treatment. AP: review of the charts. RR: patient recruitment. MP: corrections.

## Conflict of Interest Statement

The authors declare that the research was conducted in the absence of any commercial or financial relationships that could be construed as a potential conflict of interest.
